# Keeping it fresh: ribosomal protein SA sustains sarcomeric function via localized translation

**DOI:** 10.1172/JCI181996

**Published:** 2024-07-01

**Authors:** Abigail Nagle, Michael Regnier, Jennifer Davis

**Affiliations:** 1 Department of Bioengineering,; 2 Center for Translational Muscle Research,; 3 Institute for Stem Cell and Regenerative Medicine,; 4 Center for Cardiovascular Biology, and; 5 Department of Pathology and Lab Medicine, University of Washington, Seattle, USA.

## Abstract

Mechanical stress from cardiomyocyte contraction causes misfolded sarcomeric protein replacement. Sarcomeric maintenance utilizes localized pools of mRNAs and translation machinery, yet the importance of localized translation remains unclear. In this issue of the *JCI*, Haddad et al. identify the Z-line as a critical site for localized translation of sarcomeric proteins, mediated by ribosomal protein SA (RPSA). RPSA localized ribosomes at Z-lines and was trafficked via microtubules. Cardiomyocyte-specific loss of RPSA in mice resulted in mislocalized protein translation and caused structural dilation from myocyte atrophy. These findings demonstrate the necessity of RPSA-dependent spatially localized translation for sarcomere maintenance and cardiac structure and function.

## Sarcomeric protein translation and turnover

**Figure 1:**
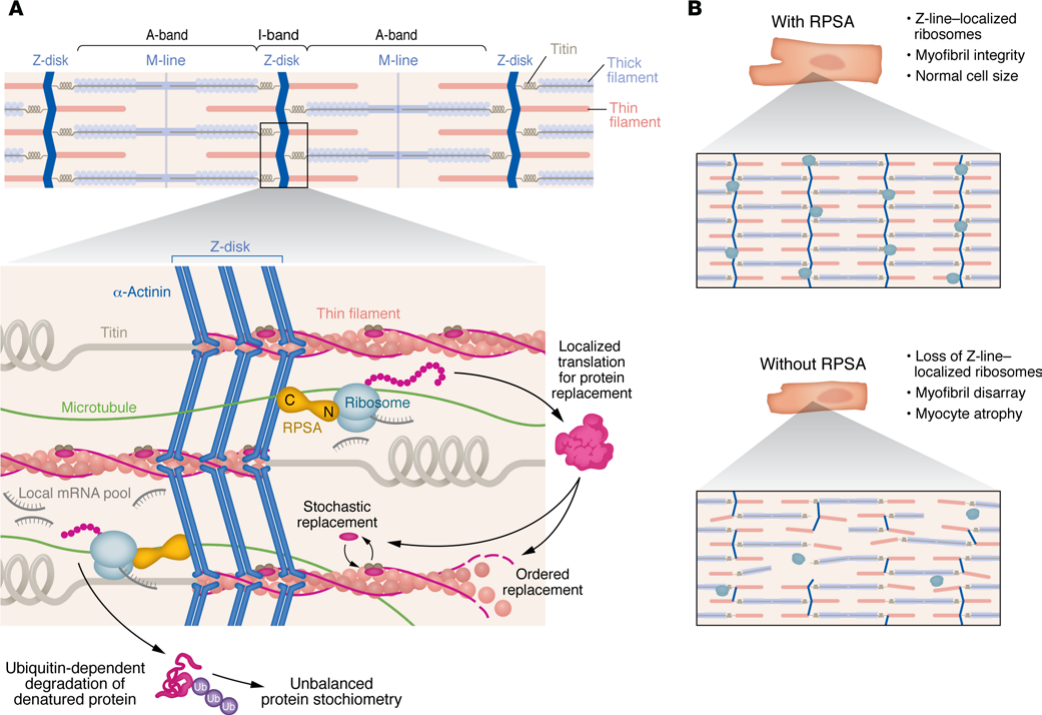
RPSA is important for maintenance of cardiac cells and heart tissue. (**A**) RPSA serves as an essential component for localized protein translation within the sarcomere. It localizes to the Z-line where it is involved with tethering ribosomes to Z-lines within myofibrils. RPSA depends on the microtubule network for its localization to the ribosomes. Structurally, the C-terminus of RPSA associates with the α-actinin–containing Z line while the N-terminus interacts with the ribosome. (**B**) Local translation at the Z-lines is important for protein replacement within the sarcomere and for maintaining myofibril integrity. Loss of RPSA leads to loss of sarcomeric protein translation, loss of ordered sarcomere structure, and myocyte atrophy.

The cardiomyocyte’s basic force-generating unit is the sarcomere. Sarcomeric proteins misfold, denature, or become dysfunctional over time due to persistent mechanical stress. These worn-out proteins must be recognized as nonfunctional and get efficiently replaced while cardiomyocytes continue to contract and generate the forces needed to pump blood each beat. Any disruptions in sarcomere protein quality control can lead to cardiomyocyte dysfunction and ultimately cardiomyopathy (1). A proper balance between protein synthesis and degradation prevents newly synthesized proteins from accumulating in the cytosol, which could impact contractile function and energy utilization efficiency. This balance also permits the efficient replacement of old myofilaments with new ones. Myofilament protein replacement half-lives are highly variable and range from 3 to 5 days for troponin subunits and 7 to 10 days for actin and tropomyosin, while myosin proteins exchange as fast as 48 hours (2, 3). Another variable is the spatial replacement of sarcomeric proteins, in which the proteins comprising the troponin complex undergo stochastic replacement within an intact filament, while others like tropomyosin undergo ordered replacement following actin filament polarity (4). It is still unclear how these processes are regulated, but previous work from the Kehat lab suggests that three location-dependent features — all localized to the sarcomere — drive sarcomeric replacement: large pools of sarcomeric mRNA templates, ribosomal protein translation machinery, and ubiquitin ligases for degradation. Indeed, mRNAs and ribosomes localize to the sarcomere to translate proteins directly at the structure of interest (5), and the replacement of sarcomeric proteins like titin requires a soluble pool of the protein produced by localized ribosomes (6). Trafficking of mRNAs and protein translation machinery to sarcomeric locations relies on the cytoskeleton. For instance, microtubules control localized protein synthesis in cardiomyocytes, and, when the microtubule network grows, protein translation increases and cardiomyocytes undergo hypertrophy (7). How turnover-induced changes in myofilament stoichiometry are sensed and regulated, as well as how translation of myofilament proteins is coordinated with spatially explicit incorporation of the newly synthesized products, remains unknown (Figure 1). There must be a mechanism by which a cell regulates mRNA translation, independent of gene regulation, to accommodate the variable replacement rates of sarcomeric proteins, given that mRNA concentrations do not correlate with protein expression of sarcomeric proteins or structure (5, 8).

## Z-line localization of ribosomes and translation

In this issue of the *JCI* some of these unknowns were addressed by Haddad et al., who identified the localized Z-line interactome (9). In their article, the authors utilized a BioID2 proximity-labeling approach in neonatal rat ventricular myocytes (NRVMs) using Cypher (LDB3) as bait. Many structural and ribosome-related proteins at the Z-line niche were identified, with ribosomal protein SA (RPSA) showing significant enrichment (9). Notably, RPSA localized to either side of α-actinin–containing Z-lines. This study differs from a Z-line proximity-labeling study by Ladha and colleagues, who used an endogenously fused BirA*-ACTN2 construct in stem cell–derived cardiomyocytes (10). Although RPSA was not found in the Ladha study, Haddad and colleagues postulated that this difference in protein niche may be due to the difference in NRVMs versus stem cell–derived cardiomyocytes or to spatial neighborhoods, as ACTN2 and Cypher reside in spatially distinct Z-line locations, with RPSA residing more toward either Z-line edge. Another confounding factor not discussed by Haddad et al. relates to the methods used between the two studies. The proximity-labeling method used by Haddad et al. may have revealed additional low-affinity relationships due to usage of an overexpressed Z-line protein, as opposed to the methods used by Ladha et al. that utilized an endogenously tagged protein. Nevertheless, Haddad et al. showed that, in vitro, RPSA was necessary for sarcomeric alignment and localization of ribosomes and nascent peptides (9).

Reportedly, RPSA has dual functions, as a nonintegrin laminin receptor and as a component of the 40S ribosome, and some authors have hypothesized that the N-terminus binds ribosomes and the C-terminus binds laminin, a component of the extracellular matrix (ECM) (11). The sites of Z-line attachment to the ECM, called costameres, are essential for myofibril nucleation and formation (12). As costameres experience a different combination of forces compared with other Z-lines, there are presumptive differences in the protein maintenance. Haddad et al. show localization of RPSA throughout the cell, so an important continuation of this work will be to tease out subcellular Z-line niche differences in translation regulation.

## In vivo deletion of localized translation causes dilated cardiomyopathy

To date most studies of localized protein translation have been performed in cultured rodent or human stem cell–derived myocytes, but Haddad et al. (9) made a major scientific leap by experimentally demonstrating that RPSA-dependent localization of sarcomere protein translation decreased cardiac function and caused ventricular dilation in mice that had a loss of RPSA function, either globally or in a cardiac-specific mosaic condition. To achieve mosaic deletion of RPSA, Cas9 nuclease–expressing mice were transduced with global or cardiac-specific AAV-expressing RPSA-targeting sgRNAs, generating a mutation of the RPSA gene. In the cardiac-specific mosaic knockout, this dysfunction was not seen until the mice reached six months of age. Fascinatingly, the myocytes that received the *Rpsa*-targeting sgRNAs were smaller than control myocytes, and the unedited myocytes in RPSA-mosaic mice hypertrophied in length and width relative to control myocytes, presumably as compensation for the loss of myocyte volume in the edited cells. Importantly, the authors showed no edit-induced cell death occurred as the mice aged. The lack of growth of edited cells was accompanied by increased expression of markers of the fetal gene program, decreased sarcomeric protein translation, and decreased global protein translation. Although global protein translation was decreased, only sarcomeric proteins had a lower protein expression level compared with that of control mice, revealing the key finding that RPSA-associated ribosome translation at the Z-line was most responsible for the translation of sarcomeric-specific proteins. Finally, Haddad et al. (9) showed a trend of decreased RPSA expression in mice with a MYPBC3 mutation causing hypertrophic cardiomyopathy (HCM), yet those mice showed no difference in subcellular RPSA localization. These data indicate that RPSA function is upstream of pathological sarcomere effects and that the disruption of RPSA-mediated localized translation negatively affects cardiomyocyte structure and function.

Though many have postulated that Z-line–localized protein translation is essential for sarcomere structure, Haddad et al. (9) provide direct evidence that loss of Z-line translation specifically negatively affects sarcomere content in cardiomyocytes. As previous work by this group and others has shown that the cytoskeleton is critical for localized translation (5, 7), Haddad et al. (9) demonstrate that nocodazole- or colchicine-mediated dissolution of the microtubules prevents RPSA peripheralization, which suggests that RPSA is the key driver of the dependence of localized translation on the microtubule network. Furthermore, the C-terminus of RPSA, previously known as a laminin receptor, is sufficient for Z-line localization. Collectively, these data support a model whereby RPSA maneuvers along microtubules to position its C-terminus on the Z-line and the N-terminus on ribosomes to translate sarcomeric proteins locally (Figure 1).

## Conclusion and implications

The regulation of sarcomere maintenance is essential for a broad array of applications for healthy heart function. Not only do mutations in proteins involved in sarcomere protein degradation and localized translation cause cardiomyopathies (9, 13), but they also result in myofilament protein degradation during heart events such as ischemia/reperfusion, which is further detrimental to cardiac output (14). Haddad et al. (9) identified RPSA as a key mediator of sarcomere maintenance due to its necessity for ribosome localization to the Z-line. This study definitively proves that the spatial localization of protein translation is necessary for providing replacement sarcomeric proteins. Understanding the role of RPSA may unlock insights into treatments that regulate translation of sarcomeric proteins to prevent pathological remodeling caused by the misbalance of mechanical stress in the heart from congenital mutations or progressive heart disease.

The study by Haddad et al. is important because it increases our understanding regulators of sarcomere maintenance and provokes many outstanding questions (9). For instance, previous work from this group has highlighted the importance of balancing protein degradation and synthesis for sarcomere maintenance (5), yet the cues that indicate these processes are off-balance are unknown. In fact, the pathologically larger myocytes in the HCM mouse model indicated a trend of decreased RPSA expression in the heart, but myocytes lacking RPSA in chimeric knockouts showed atrophy. One possible explanation for this dichotomy involves a compensatory knockdown of RPSA to restrain hypertrophic myocyte growth in the HCM mice. Another possibility relates to heterogenous expression of RPSA throughout the myocyte population that in turn increases the diversity of myocyte cross-sectional areas throughout the heart. Understanding how a loss of RPSA is associated with pathologically smaller and larger myocytes is critical for understanding the role of localized translation for the progression of disease at a cellular level. Other unknowns worthy of investigation include how localized translation processes achieve the variable time scales of myofilament protein turnover and replacement, what regulates ordered versus stochastic replacement, and how misfolded proteins initiate sarcomeric maintenance versus addition. Haddad et al.’s findings have improved our knowledge of how ribosomes localize to Z-lines and highlighted the importance of localized ribosomes for translation of specifically sarcomeric proteins (9). Overall, the discovery that sarcomeric protein translation specifically depends on localized ribosomes to maintain proper cardiomyocyte structure and heart function represents an important progression in the field toward exploiting sarcomere maintenance mechanisms to prevent pathological disease progression.

## Version 1 07/01/2024

Electronic publication
